# On Diseases of the Skin

**Published:** 1847-01

**Authors:** 


Art. XVII.—*On Diseases of the Skin.* By Erasmus Wilson, f.r.s. Second Edition.—*London*, 1847. 8vo, pp. 482. With Eight Coloured Plates.

In our Number for April, 1843, we gave a critical review of the first edition of this book. Although disapproving of the arrangement adopted by the author, we spoke favorably of the production as a whole. We refer to our previous article for the particulars of our judgment on the treatise generally, and content ourselves, on this occasion, with stating that the work is very considerably improved in the present edition, and is for the first time illustrated with plates. Of these plates it is impossible to speak too highly. The representation of the various forms of cutaneous disease are singularly accurate, and the colouring exceeds almost anything we have met with in point of delicacy and finish.

